# Supplementary material for: Depression, anxiety and change in eating habits during the COVID-19 pandemic in Brazilian university students
Source: PLoS One. 2025 Jun 27;20(6):e0326856. doi: 10.1371/journal.pone.0326856 (PMC12204533; doi:10.1371/journal.pone.0326856)
Supplement: Attachment — Submitted filename: Response to Reviewers.docx [file pone.0326856.s003.docx]

**Anxiety and depression negatively affected the eating habits of university students in Brazil**

Journal Requirements

1. Please ensure that your manuscript meets PLOS ONE's style requirements, including those for file naming. The PLOS ONE style templates can be found at https://journals.plos.org/plosone/s/file?id=wjVg/PLOSOne_formatting_sample_main_body.pdf and https://journals.plos.org/plosone/s/file?id=ba62/PLOSOne_formatting_sample_title_authors_affiliations.pdf

**A**: Style requirements revised.

2. Thank you for stating the following financial disclosure:

"Funding received according to the grant n° 255937: Emergencial Action COVID-19 - Call C - Support for Research Network Projects on SARS-CoV-2/COVID-19. Funding provided by Carlos Chagas Filho Foundation (FAPERJ)."

Please state what role the funders took in the study. If the funders had no role, please state: ""The funders had no role in study design, data collection and analysis, decision to publish, or preparation of the manuscript."" If this statement is not correct you must amend it as needed. Please include this amended Role of Funder statement in your cover letter; we will change the online submission form on your behalf.

**A**: Amended Role of Funded statement added to the Cover Letter.

3. Please include your full ethics statement in the ‘Methods’ section of your manuscript file. In your statement, please include the full name of the IRB or ethics committee who approved or waived your study, as well as whether or not you obtained informed written or verbal consent. If consent was waived for your study, please include this information in your statement as well.

**A**: Full name of the IRB added; the ethics statement contains the following information regarding the written consent “*An informed written consent term was obtained from each participant via a specific field on the Google Form questionnaire*” that can be found on lines 158 and 159 of the Revised Manuscript with Track Changes.

4. Please upload a copy of Figure 4, to which you refer in your text on page 16. If the figure is no longer to be included as part of the submission please remove all reference to it within the text.

**A**: We meant to write “Table 4” and wrote “Figure 4” by accident. Corrected.

Additional Editor Comments

Introduction

1. As the results are presented by gender, I suggest considering this difference in the introduction.

**A**: We added a new mention and reference regarding gender that can be seen on lines 58 and 59 of the Revised Manuscript with Track Changes.

Methods

2. In what period, month, year and wave of COVID-19 was this research done? Did we already have a vaccine? If so, may or may not this have affected the perception of the pandemic and thus anxiety and depression or eating habits?

**A**: Our data was collected between August 6, 2020 and March 13, 2021, as stated on line 76 of the Revised Manuscript with Track Changes, a period that comprehends the second wave of COVID-19. No vaccines were available in Brazil. New text was added stating this information (lines 76 and 77).

3. No sample calculation was carried out? What is the sampling power to capture differences in relation to population size?

**A**: The sampling power to capture differences in relation to population size was 0.8, the sample size using a proportion of 50% and an error of 0.05 was 384. This information was added on lines 74 and 75 of the Revised Manuscript with Track Changes.

4. The authors must consider that they are two instruments for the epidemiological screening of anxiety and depression and not for the clinical diagnosis of these conditions.

**A**: We made sure to point out in the methodology section of the text that our instruments are used to investigate the presence of self-reported anxiety symptoms. No mentions of clinical diagnosis of anxiety and/or depression were made throughout the text.

5. The sociodemographic variables that make up the article, especially in table 1, were not described in the methods section. For example, area of study and what corresponds.

**A**: After careful consideration the authors have decided to remove the “knowledge area” variable from Table 1, since this variable wasn’t used on our analysis or anywhere else in the text. Regarding the other sociodemographic variables, we identified that “race” and “weight status (BMI)” may elicit some doubt in the reader and further explanation was provided on lines 117 to 120 of the Revised Manuscript with Track Changes.

Now, weight status was defined as BMI classification of the WHO and race/ skin color was defined in agreement with the Brazilian Bureau of Census.

6. Was the research approved by a research ethics committee in Brazil? What number indicates this approval?

**A**: Yes, our study was approved by the Research Ethics Committee of the Institute of Social Medicine at the State University of Rio de Janeiro, and it received approval based on opinion number 4,189,209. This information was added on lines 154 to 156 of the Revised Manuscript with Track Changes.

Discussion

7. Can it be said that mental health problems were identified with the questionnaires administered? Or would it be better to talk about the symptoms of the conditions being researched?

**A**: Mental health problems can not be identified with the questionnaire administered, only symptoms. We have removed this statement on lines 247 and 248 of the Revised Manuscript with Track Changes.

8. There are other limitations that the authors did not mention: Information, memory, confirmation bias. This is tracking data. Sample size. University students from a single state in Brazil, with a financial standard that may define a dietary pattern that may be different from the general and non-university adult population.

**A**: Since the study was conducted during the first waves of the pandemic, the auctors don’t believe that memory and confirmation bias are an issue. The reviewer made a good point on the possible lack of representability of our sample and we added a third limitation pointing this out, located between lines 302 and 306 of the Revised Manuscript with Track Changes. Also, we added the argument used by the editor.
