# Supplementary material for: Depression, anxiety and change in eating habits during the COVID-19 pandemic in Brazilian university students
Source: PLoS One. 2025 Jun 27;20(6):e0326856. doi: 10.1371/journal.pone.0326856 (PMC12204533; doi:10.1371/journal.pone.0326856)
Supplement: Attachment — Submitted filename: Response_to_Reviewers_auresp_2.docx [file pone.0326856.s004.docx]

**Depression, anxiety and change in eating habits during the COVID-19 pandemic in Brazilian university students [PONE-D-24-00167R1]**

Dear Editor,

We are pleased to resubmit our revised manuscript now entitled “Depression, anxiety and change in eating habits during the COVID-19 pandemic in Brazilian university students” [PONE-D-24-00167R1], for consideration for publication in *Plos One*.

We would like to express our sincere appreciation to you and the reviewers for your careful reading of our manuscript and for the insightful comments and suggestions that helped improve the quality and clarity of our work.

A detailed point-by-point response to each reviewer follows below, outlining how their feedback was integrated. Also, we did a thorough English and grammar review of the contents of the manuscript, in order to increase its readability.

We believe the revisions substantially strengthen the manuscript and bring greater clarity to our contribution regarding eating behaviours, psychological symptoms, and public health challenges among university students during the COVID-19 pandemic.

We thank you again for your time and consideration and look forward to your feedback.

Sincerely,
**Marcus Vinicius Barbosa Verly-Miguel** (on behalf of all co-authors)
Institute of Social Medicine, State University of Rio de Janeiro

**Reviewer 2:**

The conclusion of the article is concise but does not sufficiently address the main findings and implications of the study. For a manuscript submitted to a high-impact journal like PLOS ONE, it is recommended that the Conclusion section be more comprehensive, reflecting the following points:

Summary of key findings: The conclusion should synthesize the main findings discussed in the paper, particularly the significant relationship between symptoms of anxiety and depression and the increased consumption of hyperpalatable foods, mediated by meal substitution with snacks. This is crucial to reinforce the importance of the discoveries.

Practical and scientific implications: The impact of the results on the mental and behavioral health of university students should be emphasized more strongly. For example, the conclusion could discuss the potential long-term implications of these eating habits for both the mental and physical health of the university population.

Study limitations: While the limitations have been addressed in the discussion, it would be important to briefly mention them in the conclusion, particularly the high non-response rate and the online data collection method, which may affect the generalizability of the results.

Suggestions for future research: Including a brief mention of future research directions could enhance the conclusion. For instance, suggesting longitudinal studies to investigate the continuity of these eating behaviours post-pandemic or applying the study to other university populations from different geographical and socioeconomic contexts.

A more robust conclusion would help better contextualize the results and provide a clearer understanding of the study’s relevance to the field of public health and eating psychology. Additionally, it could strengthen the paper’s impact on its readership.

**Response to Reviewer 2:**

We appreciate your valuable recommendation. In response, we have expanded the Conclusion section to approximately two paragraphs, addressing the key findings, practical and scientific implications, study limitations, and suggestions for future research (see lines 317–335 in the revised manuscript). Below is a summary of how each of your concerns has been addressed:

- Summary of Key Findings: We synthesised the main results of the study, emphasizing the significant association between symptoms of anxiety/depression and increased intake of hyperpalatable foods, mediated by the replacement of regular meals with snacks.
- Practical and Scientific Implications: We highlighted the potential long-term impacts for both physical and mental health and emphasized the need for university-based programmes and broader public health interventions to mitigate these maladaptive dietary behaviours.
- Study Limitations: We briefly addressed the primary limitations, including the high non-response rate and the use of online data collection, which may limit the generalizability of the findings.
- Suggestions for Future Research: We suggested longitudinal studies to assess the persistence of these behaviours beyond the Covid-19 pandemic and highlighted the importance of expanding research to more diverse geographic regions and student populations.

**Reviewer 3:**

In the process of composing and describing variables, the authors treat the self-reported manifestations of anxiety and depression symptoms as mental disorders. The reviewer considers that this expression can lead to confusion to the less experienced reader on the subject, since it would characterize the possibility of considering the presence of mental disorders by the set of answers to the questionnaires. Although it is known by the authors that this is not the main objective of their work, the reviewer is careful to characterize the possible confusion on the part of the reader. It is therefore suggested that the authors may replace the expression mental disorders with self-reported symptoms of anxiety and/or depression, or even with responses of self-reported symptoms of anxiety and/or depression.

The discussion item presents a close relationship between the objectives of the investigation and the findings in the international and national literature regarding the possibility of explaining the characteristics of food choice and manifestations, whether demographic, school, gender, or even from the interference of symptomatic manifestations of anxiety and depression. The conclusion can be briefly expanded to account for the main aspects, including limitations, of the study, even if they are presented in the previous topic.

The study deals with a sensitive, important, current and relevant theme in the context of human development, collective health, nutritional health as well as social processes related to the university environment, in a specific period that was the COVID-19 pandemic. For these reasons, the reviewer reinforces the need for and importance of the manuscript being clear and not generating doubts, while encouraging authors to rectify it.

**Response to Reviewer 3:**

Mental Disorders: Thank you for highlighting this concern. In response, we have revised the manuscript to replace all instances of the term “mental disorders” with either “self-reported symptoms of anxiety and/or depression” or “symptoms of anxiety and depression”, as appropriate. This change was made to prevent any misinterpretation regarding clinical diagnosis and to more accurately reflect the self-reported nature of the data collected. We believe this adjustment enhances the clarity and conceptual precision of the manuscript, particularly for readers less familiar with the topic.

Conclusion Section: We have expanded the Conclusion to more explicitly summarize how the study’s objectives were addressed, while also reiterating key methodological limitations (see lines 317–335). These changes aim to reinforce the study’s contributions and ensure a more comprehensive and transparent final synthesis.

**Reviewer 4:**

In this review, I was only responsible for assessing whether the authors had responded to the suggestions for changes made by previous reviewers.

I found that all suggestions were accepted and questions answered. I therefore consider the manuscript accepted for publication.

I would like to take this opportunity to also make some suggestions, only for some additional improvements. There is no need to return it to me.

Title – I suggest including a mention of covid-19.

Abstract – The sentence about the analysis method is after the results. I suggest inverting it.

Introduction – I suggest inserting the percentage value next to the numbers 3.973 and 771 (line 72)

Methods

- Sample weighting – There is no need to repeat the values 5.9 and 18.7 (lines 83 and 84)
- Questionnaire: If possible, insert a bibliographic reference in line 88, when it is mentioned that 29 food-items frequently cited in dietary surveys in Brazil were included or inform the types of foods included.
- Study variables – check if the translation of the meaning of the acronym BMI (weight status?) is correct
- Data Analysis – Press enter in the sentence that begins with ‘To assess the goodness...’ (line 145)

Results

- Standardize table formatting (see detailed item below).
- Complete the value after the decimal point in the text. It was not presented in line 182 (46%)
- There are sentences that are in this section, but that should be taken to the methods section. Lines: 204 to 210 ‘Modification indexes were used... with cakes consumption’. Or for the Discussion section – lines 196 to 201

Discussion

- There is no need to repeat the results presented previously. If they are maintained, leave only one space after the comma. Example: lines 286 – (65.24%), 287, 291, 292, 310

Conclusion – Insert recommendations for health strategies or policies that can change this reality.

References – Check if the way of presenting the references is in accordance with the journal’s instructions. There are some that contain ellipses in the presentation of the authors/co-authors. Examples: 15, 18, 20, 33 and 35.

Tables

- The titles of all tables should be complemented with the location and year.
- (,) and (.) are being used to present the percentage values. Correcting
- There are percentage values where a decimal point was not added (example Married – table 1)
- The tables need to be reformatted as tables. There are several corrections to be made. Example: dividing lines should not be used below the variables that correspond to the columns.
- It is not necessary to repeat (p=) in all the values that appear in the p-value column

**Response to Reviewer 4:**

We thank Reviewer 4 for confirming that all previous suggestions were addressed satisfactorily and for providing additional thoughtful recommendations. Below we outline how each suggestion has been considered and implemented:

- Title: We appreciate the suggestion to include “COVID-19” in the title, which now is “Depression, anxiety and change in eating habits during the COVID-19 pandemic in Brazilian university students”.
- Abstract: The current order reflects the logical flow of structural equation modelling. We presented the results first to describe the observed associations, followed by a description of the modelling approach used to support these findings. This sequence is intended to ensure clarity regarding the data patterns before introducing the analytical framework and underpins them. However, we remain open to revising this order should the editorial board prefer the conventional structure.
- Methods: Population and study design: Percentage values were added as suggested (see line 72)
- Methods: Sample Weighting: The decision to repeat these values was intentionally retained to aid reader understanding of the sample distribution and weighting rationale.
- Questionnaire: Added an extra bibliographic reference, as suggested by the reviewer (see line 90, reference 22)
- Study variables: We revised the description of BMI to ensure accurate terminology and clarity for international readers (see line 118)
- Data Analysis: A line break was added to improve readability, as suggested (see lines 147-148)

Results:

a) All tables were standardized, according to formatting guidelines.
b) Missing decimal point was added (see line 184)
c) Moving sentences: Regarding the reviewer’s suggestion to relocate sentences (lines 196–210), we chose to retain this content in the Results section to maintain analytical transparency. Presenting the use of modification indices and the decisions behind model adjustments alongside the results allows readers to follow the evolution of the analysis and understand how key methodological choices shaped the findings.

Discussion: We maintained select results in the Discussion section as a contextual reminder for readers. However, we implemented the reviewer’s formatting suggestion by ensuring consistent spacing after commas (see lines 286-294 and line 309)

Conclusion: We expanded the Conclusion to include specific and policy-oriented recommendations for universities and public health stakeholders, with an emphasis on promoting healthier food environments (see lines 317–335).

References: Thank you for bringing this matter to our attention. All references have been revised to align with the journal’s guidelines. Specifically, ellipses were replaced with “et al.” after the sixth author’s name.

Tables:

a) Location and year: We appreciate the reviewer’s suggestion, however, since the location and study year are clearly described in the Methods section, we believe repeating this information in each table title may be redundant. To maintain clarity and avoid unnecessary repetition, we kindly opted to keep the current table titles unchanged.
b) Remaining suggestions: All tables (Tables 1 through 4) were revised to incorporate the remaining suggestions.
